# A PILOT FAMILY COACHING INTERVENTION FOR PEOPLE WITH TYPE 2 DIABETES AND A FAMILY MEMBER

**DOI:** 10.1093/geroni/igad104.1932

**Published:** 2023-12-21

**Authors:** Kristin August, Kathleen Jackson, Marsha Rosenthal

**Affiliations:** Rutgers University, Camden, New Jersey, United States; Rutgers University, Camden, New Jersey, United States; Rutgers University, New Brunswick, New Jersey, United States

## Abstract

Type 2 diabetes is a common chronic condition in later life that requires substantial lifestyle changes. Most diabetes management occurs at home with the help – and sometimes hindrance – of family members. Although coaching has become a common strategy in healthcare to supplement diabetes education, few interventions have prepared family members to be coaches, despite the documented benefit of including family members in chronic disease management interventions. The overall goal of our pilot intervention was to develop and test a family coaching approach to enhance patients’ diabetes self-management while preserving relationship quality and both individuals’ well-being. Participants were recruited from local diabetes education classes and through provider referrals, and included 20 dyads in the intervention group (patient Mage=59.12) and 20 dyads in the control group (patient Mage=57.06). The intervention, involving 5 interactive group sessions (in-person or virtual) led by a facilitator, covered evidence-based material developed by the investigators on diabetes education and coaching strategies. Behavioral, clinical, emotional, and relational metrics were assessed via a survey at baseline, post-intervention, and 3 months later; intervention participants also completed a program evaluation survey and participated in a focus group. Survey findings revealed some positive effects for diabetes self-management behaviors, no effects for short-term clinical markers, negative effects for emotional well-being, and both positive and negative effects for relational well-being. Participants reported being satisfied with the program overall; they indicated learning a lot and having a positive experience. A translation and cultural adaptation of this program for Spanish-speaking populations is currently underway.

